# The Morality of Kidney Sales: When Caring for the Seller’s Dignity Has Moral Costs

**DOI:** 10.1007/s11673-023-10231-0

**Published:** 2023-02-20

**Authors:** Alexander Reese, Ingo Pies

**Affiliations:** 1 Martin Luther University Halle-Wittenberg, Faculty of Law and Economics, Grosse Steinstrasse 73, 06108 Halle (Saale), Germany; 2 Wittenberg Center for Global Ethics, Lutherstadt Wittenberg, Germany

**Keywords:** Organ procurement, Kidneys, Transplantation, Vital organ donation, Human dignity

## Abstract

Kidney markets are prohibited in principle because they are assumed to undermine the seller’s dignity. Considering the trade-off between saving more lives by introducing regulated kidney markets and preserving the seller’s dignity, we argue that it is advisable to demand that citizens restrain their own moral judgements and not interfere with the judgements of those who are willing to sell a kidney. We also argue that it is advisable not only to limit the *political* implications of the *moral* argument of dignity concerns toward a market-based solution but also *to re-evaluate* the dignity argument itself. First, if the dignity argument is to be given normative force, it must also consider the dignity violation of the potential transplant recipient. Second, there seems to be no compelling notion of dignity that demonstrates why it is morally permissible to donate but not to sell a kidney.

## Introduction

The ability to transplant a kidney from a deceased or living donor is no doubt one of the greatest achievements in the history of medicine. Numerous lives have been saved. Yet, this very achievement creates a moral problem: many more lives could be saved if potential donors were not only permitted to provide a kidney for free but also for money.

As of this writing, 95,000 patients in the United States are on the waiting list for a kidney transplant. Every year, around 9,000 patients on that waiting list die or become too sick to undergo transplantation.^1^ Meanwhile, the yearly number of patients added to the waiting list regularly exceeds the actual number of transplantations—exacerbating the shortage in kidneys and consequently inducing more preventable deaths and a thriving black market with its problems for buyers and sellers. The COVID-19 pandemic is expected to further compound the shortage since people with COVID-19 are predisposed to kidney disease (Henry and Lippi 2020). On balance, the kidney shortage is a global phenomenon that prevails despite decades of efforts with various altruism-based policies.

An argument has been made that there is an evident solution to this “organ disaster” (Beard and Osterkamp 2014): pay people for donating their kidneys. Becker and Elías (2007), for instance, estimate that payments between $15,000 and $30,000 would eliminate the waiting list within a few years. While the empirical potency of kidney markets is reaching consensus among scholars, their normative implications are a matter of dispute (Elías, Lacetera, and Macis 2017, 490). One essential field of tension is marked by the contention that although introducing (well-regulated) kidney markets would save more lives, it would also seem to violate and undermine the preservation of dignity (Gillespie 2017).

Scholars on both sides of this trade-off between saving more lives and preserving dignity have concentrated on shifting the burden of proof to the opposite camp—an indicator that the debate has not made much progress since its inception. Against this background, the purpose of this paper is to clarify the normative stance of the concern for dignity in the debate and to propose three consecutive counterarguments that challenge the status quo’s reliance on altruism in favour of a market-based solution. The overall aim is to advance the academic and public debate on the moral legitimacy of kidney sales.

We develop our line of argumentation in three steps. In the following section, we distinguish between human and social dignity and thereby reconstruct the argument by which opponents of kidney markets justify the status quo’s reliance on altruism. Next, we formulate three counterarguments against the view that concern for human (and social) dignity requires a prohibition of kidney markets. Our counterarguments build on each other. First, we assume—for the sake of argument—that a seller’s dignity might be violated by participating in a kidney market. We then argue that this has to be weighed against the dignity violation of a kidney patient who is deprived of receiving a lifesaving transplant due to market prohibition. Once this is taken into account, from a normative point of view saving lives clearly trumps minor harm reductions. Second, we argue that since unpaid kidney donations have become officially welcome, there is in fact no convincing argument why selling a kidney for a price should be regarded as a violation of a seller’s human dignity, while possible violations of a seller’s social dignity can be avoided by prudent market regulation. Third, we accept that some people find kidney sales inherently problematic but argue that there are compelling reasons why they should tolerate the mutually beneficial market engagement of kidney sellers and buyers. Finally, we conclude this paper by summarizing the main findings and providing an outlook.

Before proceeding with the following section, we would like to make clear that (and why) we concentrate on refuting the argument that a market for kidneys would be *inherently* immoral. In doing so, we abstract from pragmatic objections, that is, we explicitly assume that many thorny problems—such as harm to third parties, exploitation, misallocation, or undesirable crowding-out effects—can be solved pragmatically by finding an appropriate market arrangement that saves lives by facilitating more voluntary exchanges between supply and demand. An analogy may help to understand our point: At first sight, the idea to have locally centralized fire stations seems to be a rather bad one, since local monopolies can exert market power and extract huge rents from people in need, especially in emergency situations. But in reality, we do not observe firefighters negotiating prices before putting out a blaze. Obviously, modern societies have created institutional arrangements that protect people against such abuse. Now suppose that people called for a prohibition of fire stations on the grounds that firefighters should be protected against possible harm. In order to concentrate on comparing the dignity of firefighters with the dignity of fire victims, it makes sense to abstract from the monopoly problem. In likewise fashion, we abstract from many possible problems that could be solved via prudent market arrangements in order to concentrate on comparing the dignity of kidney donors with the dignity of kidney donees.

﻿Albertsen (2020) identified four prominent proposals of market arrangements: the unregulated current market, the regulated current market, the payment-for-consent futures market, and the family-reward futures market. Assessing the academic literature, he discusses “an inverse relationship between how ethically controversial the market models are and the increase in organs they can be expected to produce” (Albertsen 2020, 364). He holds that “the principled concerns regarding commodification seem to be the most relevant criticism across the board of models for introducing market mechanisms and incentives” (Albertsen 2020, 363). Regarding *regulated* market arrangements, we demonstrate that saving more lives via market incentives trumps principled concerns considering commodification and that it is even possible to transcend this trade-off paradigm and save more lives while protecting human (and social) dignity.

## Prohibiting Kidney Markets for the Seller’s Dignity: A Reconstruction

Following Jacobson (2007), dignity describes two distinct yet complementary meanings: human dignity and social dignity. While human dignity ascribes inherent and inalienable value to every human being by virtue of being human, often as the basis for claiming human rights, social dignity manifests itself in the interaction between individuals, groups, and societies. In contrast to human dignity, which is usually thought to be either violated or not violated, social dignity is contextual and measurable, and therefore gradually violable. Furthermore, social dignity can be classified as dignity-of-self, referring to the dignity that people ascribe to themselves, and dignity-in-relation, referring to the dignity ascribed by others (Jacobson 2007).

Opponents of kidney sales have maintained that even if well-regulated kidney markets were commendable from a consequentialist perspective, commodifying kidneys (to generate profit) would violate or even undermine the seller’s human dignity (Gillespie 2017). As such, this argument is an inherent market objection that holds irrespective of *how* kidneys are sold and bought in practice. In essence, while some proponents of market restrictions argue that dignity is always to be given precedence over competing values—such as the value of saving more lives and reducing suffering—other proponents do not accept dignity as a trumping value. In this respect, facing an assumed trade-off between preserving the seller’s dignity and saving more lives, they are open to making a devil’s bargain.

To illustrate, it suffices to consider the following three examples typical of the commodification debate:

In reply to Harris and Erin’s (2002) proposal of an “ethically defensible market in organs,” Marino et al. (2002) claim, “Any financial incentive to organ procurement […] must be avoided as it dangerously undermines human dignity by obscuring the difference between being human and marketing” (835).

Moral approval of kidney donations and condemnation of kidney sales is also articulated in several writings and speeches of John Paul II (c.f. Roth 2007). Considering organ transplant benefits to dialysis treatment, John Paul II proclaims,

[O]ne way of nurturing a genuine culture of life “is the donation of organs, performed in an ethically acceptable manner, with a view to offering a chance of health and even of life itself to the sick who sometimes have no other hope.” (John Paul II 2001, 89)

Nonetheless, he adds,

[A]ny procedure which tends to commercialize human organs or to consider them as items of exchange or trade must be considered morally unacceptable, because to use the body as an “object” is to violate the dignity of the human person. (John Paul II 2001, 90)

In a similar fashion, Cohen (2002) argues against Gill and Sade (2002), who favour a market arrangement, that it is common sense that

…the dignity of all human beings is to be respected, that the bodies of human beings are a crucial aspect of who they are, that to sell human beings or their integral body parts is to violate their dignity as human beings, and that it is wrong to allow human beings seriously to damage their own bodies in certain ways … (2002, 59)

She draws a sharp line between donating kidneys for free and selling kidneys:

If organ donation from living persons, in particular, can be justified in terms of its risks to donors and benefits to recipients, we ought to promote the altruistic donation of organs from living donors in a more communitarian fashion, rather than promote their sale. (Cohen 2002, 62)

Finally, she concludes: “Turning human organs into commodities […] is contrary to values at the core of our life together and should therefore be prohibited” (Cohen 2002, 61).

Reconstructed as a practical syllogism, the dignity argument takes the following form:

**Table:** 

Syllogism No. 1: The dignity argument in favour of a general market prohibition 1a. Human (and social) dignity should be inviolable. 1b. It is morally desirable that the state should prohibit behaviour that undermines human (and social) dignity. 2. Any person who sells one of her two kidneys degrades herself. 3. Therefore, the state should generally prohibit kidney sales.	

Drawing on the normative premise that dignity should be inviolable, this argument holds that the sale of a kidney degrades the seller and that, therefore, the state should prohibit kidney sales to protect the dignity of potential sellers.

Given this dignity argument, we formulate three critical observations.

First, this argument demonstrates vital care for the seller. As a case in point, Alpinar-Şencan, Baumann, and Biller-Andorno (2017) argue that kidney sellers, in contrast to kidney donors, “run the risk of […] being perceived as if they had a lesser worth or as if their worth was comparable to a price and that is incompatible with dignity” (198). The prohibition of kidney sales is thus assumed to prevent potential sellers from social stigmatization (Zargooshi 2001). Considering this, Rothman and Rothman (2006) emphasize that kidney sales “would point not to heroism and generosity of spirit (intrinsic reward) but to desperation and avariciousness (extrinsic reward)” (1526). We want to point out that many of these concerns do not address human dignity per se but social dignity, in particular negative reputation effects.

Second, this argument is highly paternalistic since it assumes that the person who is willing to sell one of her kidneys is making a serious mistake (Hughes 2009). She is, thus, expected either to undervalue the sale’s impact on her dignity or to overvalue the additional income made by the sale. In other words, the paternalistic stance here assumes that the potential kidney seller would be better off if she was not permitted, against her own will, to sell one of her two kidneys on a voluntary basis (Rippon 2014). Such a paternalistic approach is logically possible, but since the idea of classic liberalism plays a pivotal part in Western transplant ethics, paternalistic arguments that override individual sovereignty surely need to be backed by empirically strong and morally convincing reasons.

Third, while this dignity argument intends to protect potential kidney sellers, that is, persons assumed to be degraded, the unintended consequences of the prevalent market prohibitions have been disastrous. On the one hand, the number of preventable deaths is high. Following McCormick, Held, and Chertow (2018), the cumulative number of premature deaths in the United States due to the kidney shortage from 1988 to 2017 amounts to 982,000 human beings, expecting an additional 465,000 during the next ten years. On the other hand, potentially willing sellers are deprived of the opportunity to gain additional income—an option they subjectively take to be their best one facing the relevant alternatives. This means that the laws prohibiting kidney markets have come into existence—and persist today—because some people are, in fact, willing to enter market exchanges and others do not want them to do so (Roth 2015, 197). Thus, this prohibition results from moral intolerance.

## Criticizing the Status-Quo Reliance on Altruism

Our criticism of the status quo’s reliance on altruism rests on the observation that while today some kidneys are donated for free—or more precisely, donated without generating a profit for the donor—this number is by far insufficient to meet demand. Furthermore, basic economic reasoning leads to the insight that the kidney shortage can be relaxed or even solved if kidney donors were not only permitted to donate for free but also to sell one of their kidneys for a market price that reflects relative scarcity. Therefore, we do not focus our argument on criticizing the shortcomings and inefficiencies of the current altruistic scheme but instead concentrate on challenging the arguments favouring a strict market prohibition.

**Figure 1:**
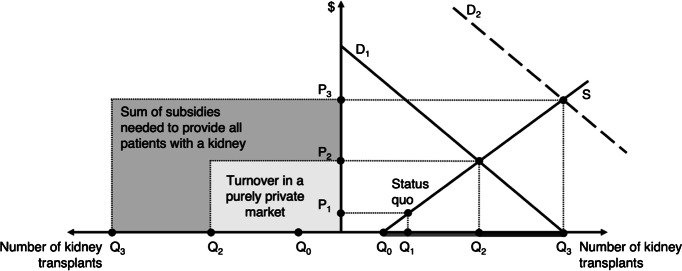
The current kidney shortage versus alternative market allocations (source: own presentation)

To make our arguments more transparent, it is helpful to use a market diagram as shown in figure 1. Here, the abscissa refers to the number of kidneys supplied for transplantation (Q), whereas the ordinate refers to the price (P) per organ. The demand curve (D_1_) has a negative slope, and the supply curve (S) has a positive slope. Under the status quo reliance on altruism, the original approach was to pursue a zero-price policy, leading to a supply of Q_0_ kidneys. In recent years, however, it has become common practice to compensate donors for income losses and to cover their medical and travel costs related to the transplantation. This means that the price has in fact risen from zero to P_1_. Currently, Q_1_ kidneys are supplied. In comparison, higher prices would lead to different allocations. In a free-market regime, the market-clearing price would equal P_2_, balancing supply and demand at quantity Q_2_. This would facilitate (Q_2_ – Q_1_) additional transplantations—and save many lives. Furthermore, in a market with health insurance (and/or government subsidies), it would even be possible to save more lives. Via financial support indicated by the dark grey area, the demand curve could be shifted to the dashed line (D_2_). This would accomplish (Q_3_ – Q_2_) further transplantations. The corresponding market price would be P_3_.

Following Bastiat (1850), it is important to distinguish between what can be seen at first sight and what can only be seen with the help of second thoughts. That which can be seen with this market diagram is the principal reason a market-based solution in the United States would be expected to prevent 5,000 to 10,000 deaths per year and to reduce the suffering of 100,000 more patients on dialysis (Held et al. 2016). That which is unseen are various other potential benefits that challenge the moral legitimacy of the current practice of relying on altruism and not on market incentives.

Following Held et al. (2016), a government compensation programme for kidney donors could provide the following additional benefits.

1. Poor Americans and African Americans are overrepresented on the kidney waiting list (cf. Tarver-Carr et al. 2002) and thus would, in particular, benefit from a compensation scheme. 2. Since transplant candidates no longer would wait for almost five years to receive a kidney transplant, the chances of successful transplantation would improve. The underlying reason is that the longer a patient is waiting for dialysis, the higher the chance that her kidney transplant might fail (Meier-Kriesche and Kaplan 2002). 3. The resulting larger pool of kidney transplants would make it easier to ensure the medical compatibility of donors and recipients, again increasing the chances of successful transplantation. 4. In case the first kidney graft fails, a second kidney transplant would be readily available, saving the lives of transplant candidates who currently do not get a second chance. 5. The additional amount of kidney transplants would more than triple the yearly net welfare gain for society, from US$20 billion per year to US$66 billion per year. The underlying reason is that dialysis is almost four times as expensive per quality-adjusted life year as a kidney transplant. 6. Incentives for Americans to participate in transplant tourism or the black market would cease (cf. Becker and Elías 2007). 7. The overall proficiency of kidney transplants would increase since kidney transplant centres could perform kidney transplants more frequently.

### First Counterargument: The Violation of the Kidney Patient’s Dignity

In this section, we provisionally accept—for the sake of argument—the premise that a person who sells one of her two kidneys necessarily degrades herself and that she thus violates her human (and maybe also her social) dignity.

Kidney sellers usually happen to be poor, so they are in danger of selling one of their kidneys out of desperation (Delmonico 2015). However, this is not to say that they are coerced to sell a kidney but that poor donors regard the option to sell a kidney as preferable compared to the relevant alternatives of generating additional income. Nonetheless, they may experience a violation of their social dignity. As Koplin (2015, 115) contends, “while employment generally promotes social standing and self-worth, the evidence from existing markets suggests that most sellers experience shame, stigma, and social isolation following the transaction.” He continues that the sellers “experience kidney sales as degrading rather than empowering” (Koplin 2015, 115). This contention accords with the concern that human organs and their donors might be “treated like fenders in an auto junkyard.”^2^

Against this background, we would like to formulate the following counterargument: if a society of citizens ascribes normative force to the moral concern of preserving human (and social) dignity—and deems it significant enough to become a binding law—then society also should adequately respect the recipient’s dignity.

Kidney patients are not exempt from experiencing social stigmatization, shame, and social isolation. They can experience shame and degradation when asking family members and friends to provide a kidney. Deprived of any opportunity to save their lives by legally buying a kidney, they may feel neglected and mistreated as second-rate citizens or human beings of lesser worth, whose right to live is endangered by other people’s moral concerns (or maybe even prejudices). Finally, kidney patients can experience social stigmatization (and also criminalization) when looking for a lifesaving transplant on the prevalent black markets, which bear witness to the fact that family members, friends, and strangers are often unable or unwilling to make a life-saving donation on purely altruistic grounds.

There are many descriptions of people who have sold, but relatively few descriptions of people who have bought, a kidney in a black market. A subjective insight is provided by Willi Germund (2015), a journalist and kidney patient who described in detail how he had, after a long trial, received a kidney transplant from the black market. Regarding his decision whether to wait in hope for a kidney one day or to venture onto the black market, he writes the following: “Personally, I must soon admit to myself in all honesty that I am only conducting a sham debate with myself. The immense horror of life on dialysis and the fear of waiting for many years are so enormous that the moral qualms of buying a kidney are regularly defeated by my inner considerations” (Germund 2015, 50–51, own translation). Using language like the auto junkyard metaphor, it is violating the human (and social) dignity of a person who faces a low probability of receiving a transplant in due course that she has to rent a billboard publicly stating, “I need a kidney.”

Consequently, the trade-off at hand is not preserving the seller’s dignity versus saving lives; rather, it is preserving the seller’s dignity versus preserving the patient’s dignity who, due to the moral prejudice of third parties, is not provided with a donor’s kidney as early as possible and whose life is thus wilfully endangered. In effect, therefore, we agree with Ng (2019, 62) that “[s]ympathy should be directed to both sides.”

Taking *both* the seller’s *and* the recipient’s dignity into consideration, we hold it necessary to deviate from the status quo reliance on altruism, which focuses one-sidedly on the seller’s dignity alone. In this regard, we argue that the factual harm to the selling person and the possible violation of her dignity are—from a *moral* perspective!—more than offset by the factual harm and the factual violation of dignity for the receiving person, since it is her *life* that is literally at stake.

On the one hand, “[t]he sale of organs is essentially rooted in the urge to survive” (Kishore 2005, 365). As laid out earlier, the prohibition of kidney sales leads to five to ten thousand avoidable deaths per year in the United States alone. Considering that human dignity ascribes every person “a right to live as if his or her life matters and to be treated ‘as a person’” (May and Daly 2019, 129), a dead person has definitely lost her predisposition to live with and promote dignity. The kidney patient involuntarily dies *because* of the imposed market prohibition.

On the other hand, the kidney seller voluntarily decides whether she opts to sell one of her kidneys at a market price. Concerning this notion of *voluntariness*, Kiener (2021) argues that the voluntariness to provide a kidney is not identical with the willingness to provide a kidney: “﻿Whereas some people feel very gratified if they can donate and help their child, others are extremely frightened and secretly hope not to be compatible. In interviews, the latter admit to be ‘scared to death’ and ‘terrified all the way down’” (Kiener 2021, 1). Just as the experience of reluctance and pressure from social expectations does not undermine autonomy, he holds that it does not undermine the voluntariness of consent either. In line with this argument, we conclude that insofar as people can donate or sell autonomously, kidney donations *and* sales are compatible with the ethical condition that the voluntariness of consent is integral to legitimizing transplantation.

In addition, the desperation of kidney patients to receive a transplant sometimes leads to stigmatization and experience of shame by family members who might be able to donate a kidney but are too reluctant to do so. Referring to the study by Scheper-Hughes (2009, 2007) on the tyranny and terror of the gift, for instance, both donations and sales can stem from substantive social pressure. Legalizing kidney sales would reduce the pressure on family members to donate a kidney and accordingly their risk of stigmatization and experience of shame in denying the request for a lifesaving transplant. Kidney markets therefore protect not only the dignity of recipients but of (potential) donors as well.

Reconstructed as a practical syllogism, our first counterargument looks as follows.

**Table:** 

Syllogism No. 2: Counterargument concerning the violation of the kidney patient’s dignity 1a. Human (and social) dignity should be inviolable. 1b. It is morally desirable that the state should prohibit behaviour that undermines human (and social) dignity. 2a. Any person who sells one of her two kidneys degrades herself. ∆2b. However, any person’s human (and sometimes social) dignity is violated who, due to the moral prejudice of third parties, is not provided with a donor’s kidney as early as possible. ∆2c. The dignity violation of those not provided with a donor kidney, due to the moral prejudice of third parties, is more significant than the dignity violation of those who sell one of their two kidneys. 3. Therefore, the state should generally allow kidney sales.	

Drawing on this syllogism, the concern for human (and social) dignity for the seller *and* the recipient (and potential donors) points to a market-based solution. This line of thought can be found, for example, in Satel (2008, 78), who concludes:

﻿[J]ust as “dignity” is invoked as a reason to oppose donor compensation, it can be seen as a potent justification for supporting it, because compensation promotes vital features of human dignity as commonly understood: the advancement of freedom [namely, the individual’s freedom to refuse renumeration], the amelioration of suffering, and the preservation of human life.

### Second Counterargument: The Inconsistency of the Dignity Objection

Having discussed the assumed trade-off between the seller’s and the recipient’s dignity, we now want to turn to another aspect of the debate. Here, our counterargument runs as follows: we cannot find a compelling normative notion of dignity that demonstrates why it is morally permissible or even praiseworthy (which most market opponents accept) to provide a kidney for free but not for money.

This lack of consistency is problematic for at least two reasons. First, facing significant shortages in kidneys for transplants, the stakes in legalizing or still prohibiting kidney markets are high. Thus, making sound moral judgements is fundamental. Second, the concern for human (and social) dignity plays a salient role in the commodification debate. If market objections based on assumed violations of dignity reveal themselves as merely weak, it would be imperative from a moral perspective to consider implementing market-based solutions. In this section, we challenge the contention that selling a kidney degrades the seller by addressing the (in)consistency of the normative notion of human (and social) dignity and its social contingency across time and culture.

(1) Regarding the (in)consistency of the notion, it is, first, striking that while the preservation of human dignity is a central principle in the ethics of transplants, it is seldom adequately defined.

To illustrate, the Declaration of Istanbul from 2008 opposes commercial organ trade because it is assumed to “violate the principles of equity, justice, and respect for human dignity” (The Declaration of Istanbul on Organ Trafficking and Transplant Tourism 2008, 1228). The Council of Europe Convention against Trafficking in Human Organs emphasizes in its preamble the assumed fact that “trafficking in human organs violates human dignity” (Convention against Trafficking in Human Organs 2015, 1). Finally, the Human Dignity and Bioethics report by the President’s Council on Bioethics places—as the report title indicates—dignity at the centre of attention for moral orientation. All three cases accentuate the importance of dignity while leaving their content vague.

Against this background, one familiar notion of dignity used by market opponents to make a prima facie case against the sales of kidneys is Kant’s categorical imperative to “use humanity […] always at the same time as an end, never merely as a means” (Kant 1785, 41).

Along these lines, Kantians may argue that removing healthy parts of a body contradicts the merely means principle, irrespective of whether the kidney is sold or given for free (cf. Cherry 2005, 133–136):

[D]isposing of oneself as a mere means to some discretionary end is debasing humanity in one’s person (*homo noumenon*), to which the human being (*homo phaenomenon*) was nevertheless entrusted for preservation. To deprive oneself of an integral part or organ (to maim oneself)—for example, to give away or sell a tooth to be transplanted into another’s mouth, or to have oneself castrated in order to get an easier livelihood as a singer, and so forth—are ways of partially murdering oneself. (Kant 1797, 190)

In a similar fashion, Morelli (1999, 320) argues that the sale of a kidney “is done for the receipt of the money to be obtained,” and therefore, “selling oneself or part of oneself is always treating oneself as a mere means.” He, nonetheless, points out that donating a kidney would not violate the Kantian principle if the donation was done for “beneficent purposes” (320), for example, for the purpose of saving another’s life.

Considering this, it is unclear why selling a kidney is *always* treating oneself as a means and, particularly, why it is always treating oneself as a *mere* means. For example, we might assume a regulated market in which only wealthy people are eligible to sell one of their kidneys. In such a market, it is realistic to assume that at least some people do not intend to sell out of desperation or mere self-interest but that their behaviour is grounded in the idea of mutual benefit. The kidney is sold to save another’s life and to gain additional money (which may be spent for praiseworthy purposes). Hence, selling a kidney might violate the mere means principle in some cases, or even in many cases, but it certainly does not violate it in *all* cases. One cannot infer the seller’s intention from just observing a sale.

(2) Addressing the social contingency of (social) dignity, scholars have pointed out a myriad of instances in which the common moral judgement on whether something promotes or undermines a person’s dignity has changed. Adam Smith (1776, 209) famously wrote—referring to the exorbitant rewards of players, opera singers, and opera dancers:

There are some very agreeable and beautiful talents of which the possession commands a certain sort of admiration; but of which the exercise for the sake of gain is considered, whether from reason or prejudice, as a sort of public prostitution.

In the present day, we are likely to regard not a high payment but a low payment (and, in particular, no payment at all) as a form of disrespect for their talent and commitment to the excellence of the beautiful art (laying aside the suspicion that employers might have other—non-altruistic—reasons not to pay). We note a similar judgement regarding the payment of teachers and nurses. Most people today not only see it as acceptable to pay them (well) but even as necessary to acknowledge their services. Reforming canonical anti-usury law is another case in point. Along these lines, Zelizer (2011) documents moral change, both with regard to the social meaning of money and with regard to the valuation of human lives, for example, the initial moral pushback against life insurance during the nineteenth century.

Irrespective of the change in moral judgements, similar activities today are judged differently. Against this background, Roth (2007, 42) highlights that the practice of dwarf tossing—a practice in which small people are thrown by large(r) people—actually leads to different moral judgements. While it is legal in the United Kingdom, the French Council of State prohibits dwarf tossing on the grounds that it “was an attraction that affronted human dignity.”^3^ One might not only call into question whether the prohibition in France takes the right decision in the assumed trade-off between preserving dignity and allowing the free choice of employment (as it was, in fact, a French dwarf who sued in court to be allowed to continue his work). One might also ask, as Roth did, why dwarf tossing is undermining human dignity but wife carrying—a practice where men race while carrying a female teammate—is not (Roth 2007, 43). Perhaps, it becomes one day a matter of dignity whether the winning team wins the wife’s weight in beer, as is standard practice.

Compensation for organ donation is no different. We just became witnesses to a recent shift in the normative notion of dignity (although dignity is, again, not specifically defined). As noted in an earlier part of this paper, the Declaration of Istanbul from 2008 opposes commercial organ trade because it is assumed to “violate the principles of equity, justice, and respect for human dignity” (“The Declaration of Istanbul on Organ Trafficking and Transplant Tourism” 2008, 1228). The subsequent Declaration of Istanbul from 2018 formulates as one of its principles, however, that “[o]rgan donation should be a financially neutral act” (Declaration of Istanbul 2018, 3). Now, compensation for income losses, as well as medical and travel costs related to a kidney donation, is deemed morally permissible, perhaps even praiseworthy. The still-missing shift from “financial neutrality” to “financial profit” seems small, a matter of quantity, not of quality.

We want to further challenge the (missing) consistency of the dignity objection with the following argument. Kidney exchange programmes—where the market transactions work entirely without money—are legal in the United States, Spain, and the Netherlands. In Germany, however, such programmes are illegal because they fail to maintain a mandatory close real relationship between donor and recipient and because it might be a hidden “organ deal” (Wissenschaftliche Dienste 2017). Paying plasma donors is illegal, thus setting moral limits on markets but filling the resulting shortages from the United States and other places where donors are paid is deemed appropriate. Finally, it is considered a violation of human dignity if a father sells one of his two kidneys to finance a medical operation that saves his daughter’s life, but it is appropriate if he saves his daughter’s life by giving her a kidney (Richards 1996, 403).

Again, reconstructed as a practical syllogism, our second counterargument looks as follows:

**Table:** 

Syllogism No. 3: Counterargument concerning the inconsistency of the dignity objection 1a. Human (and social) dignity should be inviolable. 1b. It is morally desirable that the state should prohibit behaviour that undermines human (and social) dignity. ∆2a. A person who sells one of her two kidneys does not necessarily degrade herself. 2b. Any person’s human (and sometimes social) dignity is violated who, due to the moral prejudice of third parties, is not provided with a donor’s kidney as early as possible. 3. Therefore, the state should generally allow kidney sales.	

Given the widespread moral acceptance of voluntary kidney donations (for free), we argue that selling a kidney does in fact *not* involve a violation of human dignity: since a voluntary donation is seen as praiseworthy and since (at least partial) compensation of costs has become a respected practice, it is only a minor, marginal step forward to increase financial rewards for donations. Furthermore, since selling a kidney involves a strictly voluntary decision, the bar is set extremely high for paternalistic arguments that claim to protect sellers against a violation of their human dignity, since this requires that third parties are better informed about the human dignity of sellers than the sellers themselves.

However, we acknowledge that a person who sells her kidney might indeed experience a violation of her social dignity. Here, our counterargument runs as follows. Given the existence of effective market regulation (and absence of undesirable consequences), there are possible cases in which a person can—in light of the contingency character of (social) dignity—sell one of her two kidneys without degrading herself. This means that social dignity violations are not necessary.

In sharp contrast, a patient who is denied the opportunity to receive a kidney is, without exception, deprived of the opportunity to preserve her life and thus definitively violated in her human (and maybe even social) dignity. We therefore hold that the strong improvements of recipients’ human (and social) dignity brought about by kidney markets is normatively superior to possible minor violations of sellers’ social dignity, while the human dignity of voluntary donors (and hence the human dignity of voluntary sellers) is not violated at all.

### Third Counterargument: A Problem of Tolerance

Modern society must continually choose between enacting normative arguments into law and the alternative of demanding citizens come to terms with their own moral sentiments and judgements to tolerate the deviating behaviours of other citizens. We accept that some people find kidney sales inherently problematic because they assume a violation of the seller’s human or social dignity. However, we argue that their normative judgement is not sufficient to become law—that is, that their normative judgement is not sufficient to legitimately restrain others from selling one of their kidneys on a voluntary basis.

To start with, consider the following three examples.

In “Hiding from Humanity: Disgust, Shame, and the Law,” Nussbaum (2004) argues that repugnance and disgust are not sufficient arguments for making law. She discusses the case of Stephen Roy Carr. In 1988, he shot two female hikers while they were making love at their campsite on the Appalachian Trail. One of the women was seriously injured, and one died at the scene. Charged at trial with first-degree murder, Carr made a plea for mitigation and argued that catching the two women making love was so disgusting and repugnant to him that it led him to commit this crime. However, the Pennsylvania judge rejected this argument on appeal, because “[a] reasonable person would simply have discontinued his observation and left the scene; he would not kill the lovers.”^4^ Drawing on this case, Nussbaum points out that the judge neither said that the murderer was not disgusted nor that the murderer’s disgust did not prompt the man to the crime. Instead, the judge stated that the action was not justified by disgust (Nussbaum 2004, 39–40).

According to canon law, Catholics are obligated to participate in the Eucharist on Sundays and other required holidays. We can think of a Catholic who profoundly believes that going to the church brings salvation. Thus, she believes that it is in the interest of her fellow Catholics also to go to church. Considering the falling number of faithfully attending church members in the United States and many other nations, should her normative judgement (and argument) be binding to others, for example, via law? One might further specify the context and presuppose that, instead of going to church, some of her fellow Catholics spend their time with activities deemed trivial, such as watching trash television.

Finally, imagine an Islamic cleric who believes that it is, for the interests of the parties themselves, unadvisable to marry someone outside one’s faith—for instance, that a Muslim woman marries a Christian man. Imagine he states something like this: “In a marriage, you share your money, body, heart, and soul. Is there something more important than sharing your religion? Such a relationship just becomes superficial and insincere.” Should his normative judgement (and argument) be binding to others, for example, via law?

In the United States and other countries that embrace a liberal tradition, political discourse sets the bar high. In all three cases, subjective moral judgement is not deemed sufficient to bind others via law. Instead, citizens are obligated to tolerate others’ ways of life—even if, as concerning the last two examples—their normative judgements intend to take their fellows’ interests into account. The point is that it makes a tremendous difference whether a person binds herself to her own moral judgement or whether she summons other persons, for instance, via law, to be bound to a norm they do not want to obey. This reasoning is in line with John Rawls’s view on toleration as the result of secular learning processes:

The religious doctrines that in previous centuries were the professed basis of society have gradually given way to principles of constitutional government that all citizens, whatever their religious view, can endorse. Comprehensive … moral doctrines likewise cannot be endorsed by citizens generally, and they also no longer can, if they ever could, serve as the professed basis of society. (1993, 10)

The lessons we have learned from religious dissent can—and should—be applied to moral dissent. Tolerance solves conflicts, especially the type of conflicts that Joshua Greene (2013, 293) characterizes as a “tragedy of commonsense morality,” that is, conflicts between groups with divergent moral convictions.

Finally, consider the following illustration.

A house is burning. Inhabitants are trapped inside. A large crowd has already gathered in front of the house. Then professional firefighters arrive. But before they can begin to rescue the inhabitants, the crowd interferes. Although each single one of them shies away from the supererogatory duty of rescuing the inhabitants, they insist on what they deem an important moral principle, holding that firefighting should be strictly reserved for lay people on a non-monetary basis. The crowd even wants to stop professional firefighters, for whom rescuing has become a (dangerous) source of income, by threating them with a prison sentence—in an attempt to save their health and dignity.

Although professional firefighters rescue lives on a regular basis, while kidney donation is a one-time event, from the perspective of tolerance, both cases are quite similar. The prohibition of kidney sales factually hinders willing kidney donors from saving a life for an additional income. Typically, those asking to prohibit kidney sales not only hinder sellers from saving lives for additional income; they also tend not to be motivated enough to provide one of their kidneys for free to save the life in question. Otherwise, there would be no shortage. We therefore conclude by analogy that as people’s moral emotions and arguments—such as a condemnation based on repugnance—is insufficient to legitimately hinder firefighters from saving fire victims’ lives for payment, a third party’s moral emotions and arguments are insufficient to legitimately hinder a kidney donor from saving a patient’s life for payment. Both condemnations lack the acknowledgement of what John Rawls (1993, 36–37) calls “reasonable pluralism”—the reflective self-limitation required by tolerance.

Formulated as a practical syllogism, our third counterargument looks as follows:

**Table:** 

Syllogism No. 4: Counterargument of moral tolerance 1. The state should allow citizens to implement moral convictions in their personal lives, but it should not sign highly contested moral judgements into law. 2. The moral judgement that the protection of human (and social) dignity requires a legal prohibition of kidney markets is highly contested (e.g., due to internal consistencies and blind spots of moral judgements). 3. The state should not prohibit kidney markets but instead require that they be morally tolerated.	

## Conclusion and Outlook

The prohibition of kidney markets rests on the moral judgement that selling one’s kidney at a price violates the human (and maybe social) dignity of a kidney seller, since it degrades the seller as a person and is likely to meet moral disapproval by third parties, thus reducing both the seller’s self-regard and reputation.

Against this background, we formulate three counterarguments (table 1).

**Table 1:** Overview of Three Counterarguments

No.	Human dignity violation?	Social dignity violation?	Counterargument in favour of market
1	Yes (for the sake of argument)	Yes (for the sake of argument)	Strong violation of recipient’s human (and possibly social) dignity in status quo trumps dignity concerns for sellers
2	No (due to voluntariness)	Not necessarily (due to contingency)	Donor benefits; Recipient benefits strongly
3	Subjectively yes (for the sake of argument)	Subjectively yes (for the sake of argument)	Third parties should tolerate voluntary kidney sales

First, we call for a broader perspective in order to enlighten the blind spot of public discourse. Even if one grants (for the sake of argument) that kidney sellers might experience a degradation of their human or social dignity, it is important to acknowledge that the dignity of kidney recipients (as well as the social dignity of potential donors under strong pressure from family members) is at stake. The social dignity of potential kidney recipients might be violated if they are forced to exert pressure on family members and might experience rejections of their donation requests. Furthermore, the human dignity of potential kidney recipients is certainly violated if they die due to laws that deprive them of lifesaving transplantations. In fact, this violation of human dignity falls in the same category as other fatalities that result from failure to render assistance. It amounts to wilfully letting people die. This is a very strong violation of human dignity. We therefore hold that a market for kidneys is preferable on moral grounds. In particular, we argue that saving the lives of kidney recipients and thus preserving their human (and social) dignity is much more important than possible violations of the human (and social) dignity of kidney sellers. The latter is in comparison at best a second-order concern.

Second, we draw attention to inconsistencies in academic and public discourse. Many critics condemn the selling of kidneys while praising (more or less voluntary) kidney donations. We hold that it is logically impossible to defend this line of contradistinction, especially if one considers societal learning processes with regard to moral judgements. The past has experienced numerous re-evaluations, from canonical usury laws and prohibitions of coffee, alcohol, and other substances to initial protests against life-insurance contracts. Given that the international community has recently agreed that it is not only legitimate but also legal to compensate the cost of donating a kidney, it is only a gradual next step (instead of breaking a taboo) to move toward a market arrangement that covers all opportunity costs a seller deems relevant. We therefore argue that a kidney market would not violate a seller’s human dignity, while it would strongly protect the human dignity of recipients. Considering the relevant alternatives, the concern for dignity points not against but in favour of kidney markets.

Third, these criticisms of blind spots and inconsistencies lead to our strongest argument: we hold that the moral judgement against kidney markets is at best controversial. Even if one disagrees with the line of thought of our two first arguments, opponents must acknowledge that the current debate has reached a state of “reasonable pluralism.” This fact calls for a moral self-limitation in the sense of applying the time-proven principle of tolerance. Modern societies have to choose whether to allow one group of citizens to hinder another group of citizens from voluntary interaction or whether instead to obligate the first group to tolerate the behaviour of the second group. In the case at hand, the principle of tolerance would save many lives and overall enhance the human (and social) dignity of potential kidney donors and, most notably, kidney recipients.

In sum, our contribution aims at advancing the academic and public debate on the moral legitimacy of kidney sales. We show that it is advisable to limit the *political* implications of the *moral* argument of dignity concerns toward a market-based solution and even to *re-evaluate* the dignity argument itself. First, we argue that if the dignity argument is to be given normative force, it also must consider the dignity violation of the potential recipient—pointing against the prohibition of market-based solutions. Second, we emphasize that the notion of dignity lacks consistency, that it is socially contingent and, thus, revisable. Third, once it becomes clear that normative attention must be directed to the seller’s dignity as well as to the recipient’s dignity (and to the dignity of potential donors under social pressure), the dignity argument loses its tendency in favour of prohibition and gives way to a “reasonable pluralism” of moral concerns, which then strongly commends the toleration of kidney markets.

After acknowledging that market arrangements for kidney transfers are not per se illegitimate, the academic and public debate could move on to tackle the thorny problems that are necessarily involved in such an endeavour. As an outlook, we would like to hint at one specific among many problematic aspects. In terms of figure 1, we can identify a minimalist and a maximalist solution: a purely private market arrangement, characterized by turnover P_2_ ⋅ Q_2_, and a market arrangement backed by social security, characterized by turnover P_3_ ⋅ Q_3_. There are good reasons to regard the minimalist solution as a form of undersupply, since poor people may lack the income to express their vital need for a kidney with a marginal willingness to pay. Likewise, there are good reasons to regard the maximalist solution as a form of oversupply, since social security may tend to provide people with extremely expensive kidneys even if they are of little use to them. Against this background, one can expect different countries choosing different paths of experimentation how to fit kidney markets into their country-specific health sector and their respective rationing schemes.

Finally, we would like to hint at an important feedback loop. On the one hand, our discussion of dignity may help open the door for relevant policy discussions on how to create well-functioning kidney markets. On the other hand, such policy discussions may have vital repercussions on the normative discourse about the legitimacy of market-based kidney exchange. For example, it might help raise public attention to the extremely unequal treatment of patients in need of kidney transplants and, say, patients in need of heart surgery. While the latter benefit from costless access to a kind of oversupply that is generally typical of social security arrangements, the former are currently deprived of even the minimalist undersupply of a purely private market. Most of them are not provided for at all. They die on dialysis, with vain hopes on waiting lists. The political decision to ban kidney markets and thus to disregard these patients’ vital interest in survival is clearly an exemption from the rule that the health sector is meant to serve people in need. To appreciate this extraordinary exception may help people better understand the involved violation not only of the social dignity but even of the human dignity of patients in need of a life-saving kidney transplant.

## Footnotes

1 Based on OPTN data

2 *National Organ Transplant Act*. Pub. L. No. 98–507. 98 Stat 2339 [1984].

3 Wackenheim v. France, Comm. 854/1999, U.N. Doc. A/57/40, Vol. II, at 179 [HRC 2002]

4 *Com. v Carr*, 580 A.2d, 1364–1365.
